# Mental Health Challenges and Workplace Risk Factors Among Sexual and Gender Minority Workers in Korea

**DOI:** 10.1016/j.shaw.2026.04.005

**Published:** 2026-05-05

**Authors:** Munyoung Yang, Hanul Kim, Keonhee Cho, Sejung Kim, Sinyoung Yang, Soyeong Park, Suejin Yeo, Hyoung-Ryoul Kim

**Affiliations:** 1 Department of Occupational and Environmental Medicine, Seoul St. Mary's Hospital, College of Medicine, The Catholic University of Korea, Seoul, Republic of Korea; 2 Legal Assistance Network for Queer Workers, Seoul, Republic of Korea; 3 Center for Gender and Occupational Health Rights of the Korea Institute of Labor Safety and Health (KILSH), Seoul, Republic of Korea

**Keywords:** Depressive symptoms, Mental health disparities, Risk factors, Sexual and gender minority worker

## Abstract

**Background:**

This study examined disparities in mental health between sexual and gender minority (SGM) workers and the general working population in the Republic of Korea and identified workplace-related risk factors for depressive symptoms.

**Methods:**

We conducted an online survey of 720 SGM workers (July–August 2024), assessing individual- and workplace-level factors and mental health outcomes. Mental health outcomes included depressive symptoms, suicidal ideation, and suicide attempts. Age-standardized prevalence ratios (aPRs) for depressive symptoms, suicidal ideation, and suicide attempts compared SGM workers with the general population using data from the 9th Republic of Korea National Health and Nutrition Examination Survey (KNHANES, 2022–2024) and the 7th Korean Working Conditions Survey (KWCS, 2023). Logistic regression identified risk factors for depressive symptoms.

**Results:**

SGM workers had worse mental health (aPR for depressive symptoms, 4.31; suicidal ideation, 3.60; suicide attempts, 4.52), higher job insecurity (aPR: 1.52), and more workplace violence (past month aPR, 5.16; past year, 27.62). Depressive symptoms were linked to workplace violence, a negative climate, poor work-life balance, low support, and job application avoidance due to SGM identity.

**Conclusion:**

SGM workers face disproportionate mental health challenges related to adverse working conditions and discrimination, highlighting the need for targeted protective strategies.

## 1 Introduction

The sexual and gender minority (SGM) population is estimated to comprise approximately 4-5% of the total population [1,2]. Compared to the cisgender heterosexual population, SGM individuals experience greater health disparities, including elevated risks for mental disorders, cardiovascular disease, gastrointestinal conditions, and certain cancers [[3], [4], [5], [6], [7]]. Among these, mental health outcomes such as depression, anxiety, and suicide attempts are particularly pronounced [8]. A meta-analysis of youth populations reported a depression prevalence of 26% (95% confidence interval [CI]: 21–32%) among sexual minority youth and 46% (95% CI: 36–56%) among gender minority youth [9]. These elevated mental health risks are attributed to minority stress stemming from discrimination and stigma, which also contributes to immune dysregulation through biological stress mechanisms [[10], [11], [12]].

There is a growing but still limited body of research on the occupational environment of the SGM population. A recent study on workplace violence against transgender individuals in the United States reported that 50% had experienced workplace violence based on their gender identity [13]. A US survey found that 34% of the SGM population reported experiencing employment-related discrimination—such as being fired or disadvantaged in hiring or promotion—and that 35% of cisgender sexual minorities and 57% of gender minority individuals reported experiencing workplace violence [14]. Discrimination against SGM individuals also affects job quality: Canadian data indicate that sexual minority workers are three times more likely to hold precarious employment than their heterosexual counterparts [15].

In the Republic of Korea, awareness of discrimination against SGM individuals has been growing, fueled by public debates over antidiscrimination legislation [16] and several high-profile incidents [17,18]. However, in the Republic of Korea, same-sex marriage is not legally permitted, and the requirements for legal gender recognition for transgender individuals remain stringent [19]. Furthermore, antidiscrimination legislation has not been enacted since its initial proposal 19 years ago, largely due to social opposition [20]. Consequently, SGM individuals in the Republic of Korea face substantial institutional and cultural discrimination. Previous Korean studies have explored how lifetime discrimination and stigma affect mental health and contribute to health disparities among SGM populations [[21], [22], [23], [24]]. However, research specifically examining the relationship between workplace discrimination, occupational conditions, and mental health outcomes remains scarce. This study aimed to assess mental health risks among SGM individuals by comparing them to the general working population and to identify work-related risk factors associated with adverse mental health outcomes.

## 2 Methods

### 2.1 Participants and data collection

The online survey was conducted over a one-month period from July to August 2024. Participants were eligible if they were Korean nationals aged 19 to 64, based on self-reported year of birth, and resided in the Republic of Korea, and had engaged in paid work for at least one hour during the past week. Participants reported sexual orientation (heterosexual, homosexual, bisexual, pansexual, or asexual) and gender identity (cisgender, transgender man, transgender woman, or nonbinary); those identifying as both heterosexual and cisgender were excluded. A total of 720 participants who completed all relevant survey items on the work environment and mental health were included in the analysis.

This study was approved following ethical deliberation by the Institutional Review Board of Seoul St. Mary’s Hospital, College of Medicine, The Catholic University of Korea (IRB No. KIRB-20240709-021). The authors confirm that informed consent was obtained from all participants and that all procedures involving human participants were conducted in accordance with the relevant national and institutional ethical guidelines, with the research completed in compliance with the Declaration of Helsinki as revised in 2013.

Survey items were developed based on nationally representative datasets, namely, the 9th Republic of Korea National Health and Nutrition Examination Survey (KNHANES, 2022–2024) and the 7th Korean Working Conditions Survey (KWCS, 2023), to enable comparisons with the general population and workforce in the Republic of Korea [25,26]. The survey link was distributed with support from several SGM advocacy organizations (e.g., via messenger platforms and banner postings on online SGM communities).

### 2.2 Questionnaire items

The survey assessed individual-level factors, including relationships with family of origin and current family (e.g., coming-out status and whether family members were supportive or phobic toward one's identity), internalized homophobia for the sexual minority population [27] or internalized transphobia for the gender minority population [28], and the highest level of education attained. Work environment factors included occupation, place of employment, employment contract type, weekly working hours, company size, tenure, labor union, monthly income, and job security (assessed as whether participants could remain in their workplace, with responses coded as a binary variable), using KWCS items.

Mental health outcomes were assessed as follows. Depressive symptoms were measured using the Patient Health Questionnaire-9 (PHQ-9), with a cutoff score of ≥10 indicating a need for clinical evaluation [29]. Suicidal ideation was identified by an affirmative response to the question: “In the past year, have you seriously considered suicide?” Suicide attempts were measured by the question: “In the past year, have you attempted suicide?”—both adapted from KNHANES.

Workplace violence was measured using KWCS items capturing experiences of verbal abuse, unwanted sexual attention, threats, and insulting behavior in the past month, as well as physical violence, sexual harassment, or bullying in the past year. In addition to analyzing each type of violence separately, two composite indicators were created to reflect the experience of any form of workplace violence: (1) having experienced at least one of verbal abuse, unwanted sexual attention, threats, or insulting behavior in the past month, and (2) having experienced at least one of physical violence, sexual harassment, or bullying in the past year.

Job stress factors—specifically lack of social support and negative workplace climate—were assessed using items from the Korean Occupational Stress Scale (KOSS) [30]. Social support was measured through four items: (1) “My supervisor helps me complete my work,” (2) “My colleagues help me complete my work,” (3) “There is someone at work who understands and sympathizes with me when I am having a hard time,” and (4) “I have colleagues with whom I can share the hardships of workplace life.” Workplace climate was assessed with four items: (1) “Company gatherings are uncomfortable,” (2) “I receive work instructions without clear standards or consistency,” (3) “The workplace atmosphere is authoritarian and hierarchical,” and (4) “I experience discrimination based on gender (male/female).” Each item was rated on a 4-point Likert scale, and median scores for Korean workers were used as the cutoff [31].

Work–life balance was assessed using KWCS items regarding the suitability of working hours for maintaining family life and social activities. Responses of “inappropriate” or “completely inappropriate” were coded as negative. Two additional items on work-related interference with personal life—(1) worrying about work during off-hours and (2) insufficient time with family due to work—were coded as affirmative if participants responded “always” or “mostly” on a 5-point Likert scale. Participants were also asked whether they had ever refrained from applying for a job due to their sexual or gender minority identity, with responses coded as “yes” or “no.”

Regarding coming-out status in the workplace, participants were asked whether they had intentionally come out to at least one person, had been unintentionally outed, or had not disclosed their identity to anyone, and whether they had experienced workplace rejection due to their SGM identity. For gender minority participants, the survey inquired about their experiences with gender-affirming hormone therapy or gender-affirming surgery.

### 2.3 Statistical analysis

Descriptive statistics were calculated using frequency analyses and chi-square tests. Age-standardized prevalence ratios (aPRs) with 95% confidence intervals (CIs) were computed using Byar's approximation [32] to compare mental health outcomes—including depressive symptoms, suicidal ideation, and suicide attempts—between the SGM sample and general population controls. The three outcomes were compared to the general population aged 19–64 from KNHANES.

The aPRs for job-related factors such as work–life interference, employment instability, and workplace violence were also calculated using KWCS as the reference. Within the SGM group, logistic regression analyses were conducted to identify factors associated with adverse mental health outcomes, adjusting for age and occupation. Odds ratios (ORs) with 95% CIs were estimated. All statistical analyses were performed using SAS version 9.4 (SAS Institute, Cary, NC, USA).

## 3 Results

Of the 981 individuals who agreed to participate, 867 met the inclusion criteria (i.e., engagement in paid work, self-identification as an SGM individual, and an appropriate age range). Among them, 720 respondents who completed items related to both the work environment and mental health were included in the analysis (Fig. 1).

**Fig. 1:**
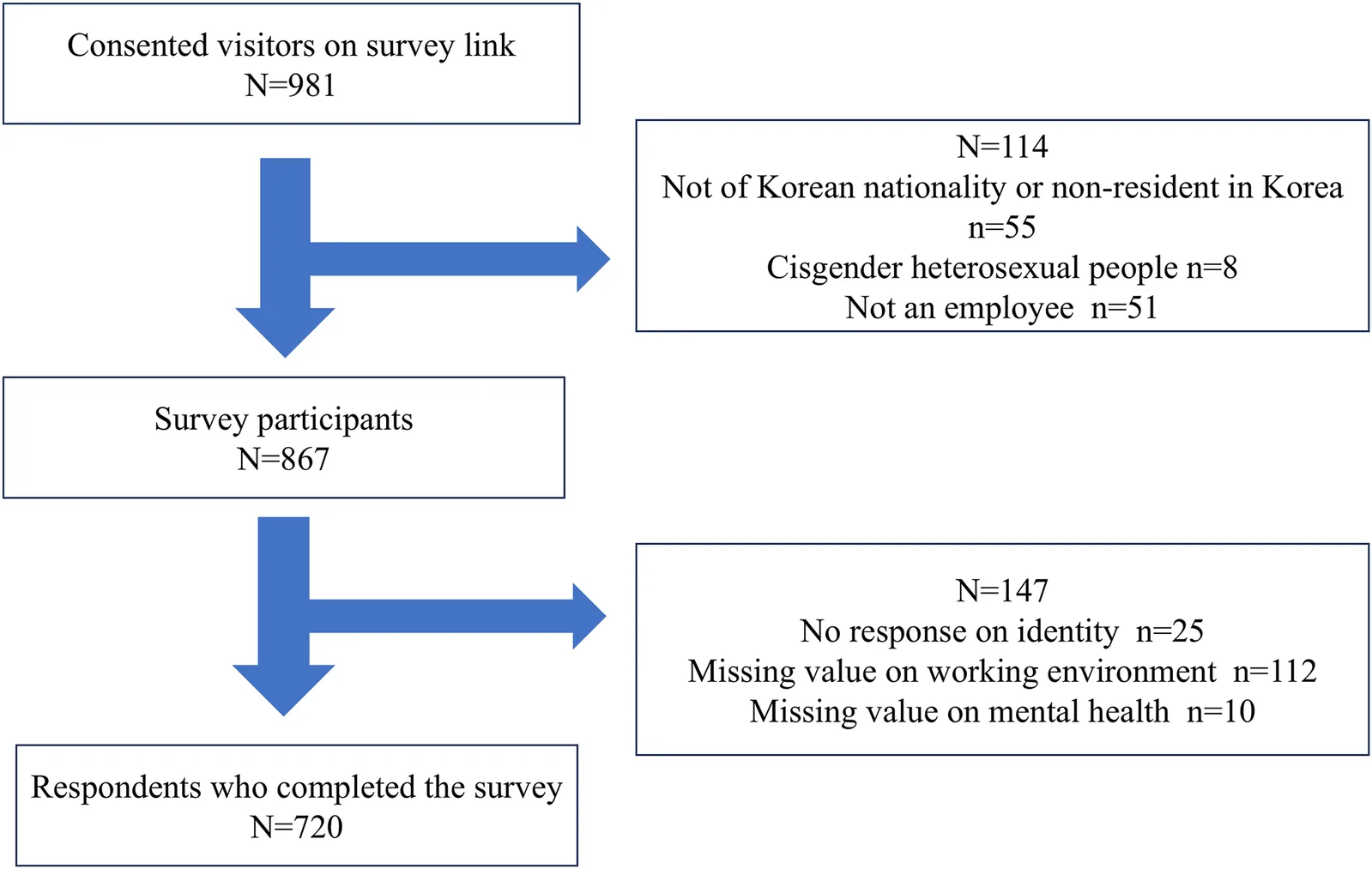
Selection of analytic population in this study.

### 3.1 Participant characteristics and prevalence of mental health outcomes

Table 1 summarizes individual-level characteristics and mental health outcomes. Among the 720 participants, 177 (24.6%) reported depressive symptoms, 129 (17.9%) reported suicidal ideation, and 22 (3.1%) reported suicide attempts. In comparison, corresponding rates in the general population were 4.9%, 4.6%, and 0.5% for the first three indicators (KNHANES). All mental health outcomes were more prevalent among SGM participants than in the general population. The prevalence of depressive symptoms was highest among nonbinary individuals (37.2%, n = 29), and suicide attempts exceeded 10% among both transmasculine and transfeminine participants—higher than that observed in other gender identity groups.

**Table 1:** Individual level characteristics of responders and the prevalence of depressive symptoms, suicidal ideation, and suicide attempt in each group

	Total	Depressive symptom		Suicidal ideation		Suicide attempt	
	*n* (% of total)	*n* (row %)	*p*	*n* (row %)	*p*	*n* (row %)	*p*
Total	720 (100%)	177 (24.6)		129 (17.9)		22 (3.1)	
Gender identity			0.042∗		0.382		<0.001∗
Transmasculine	35 (4.9)	10 (28.6)		10 (28.6)		4 (11.4)	
Transfeminine	47 (6.5)	8 (17.0)		8 (17.0)		5 (10.6)	
Nonbinary	78 (10.8)	29 (37.2)		17 (22.1)		3 (3.9)	
Cisgender men	297 (41.3)	64 (21.6)		51 (17.2)		6 (2.0)	
Cisgender women	263 (36.5)	66 (25.1)		43 (16.4)		4 (1.5)	
Sexual orientation			0.074		0.030∗		0.465
Heterosexual	9 (1.3)	4 (44.4)		3 (33.3)		1 (11.1)	
Homosexual	483 (67.1)	110 (22.8)		75 (15.6)		13 (2.7)	
Bisexual, pansexual	186 (25.8)	47 (25.3)		38 (20.4)		7 (3.8)	
Asexual	42 (5.8)	16 (38.1)		13 (31.0)		1 (2.4)	
Legal gender			0.319		0.543		0.178
Men	357 (49.6)	82 (22.9)		67 (18.8)		14 (3.9)	
Women	363 (50.4)	95 (26.2)		62 (17.1)		8 (2.2)	
Age			0.400		0.004∗		0.242
19-29	212 (29.4)	55 (25.9)		48 (22.6)		10 (4.7)	
30-39	410 (56.9)	95 (23.2)		55 (13.4)		8 (2.0)	
40-49	77 (10.7)	24 (31.2)		22 (28.6)		4 (5.2)	
50-59	18 (2.5)	3 (16.7)		3 (17.7)		0 (0)	
60-65	3 (0.4)	0 (0)		1 (33.3)		0 (0)	
Education			<0.001∗		0.023∗		0.058
High school graduate or less	111 (15.5)	49 (44.1)		27 (24.6)		7 (6.4)	
College graduate	100 (13.9)	24 (24.0)		10 (10.0)		4 (4.0)	
University graduate or more	507 (70.6)	103 (20.3)		92 (18.2)		11 (2.2)	
Coming-out status with family of origin			0.025∗		0.396		0.041∗
Someone knows and is supportive	191 (26.7)	34 (17.8)		67 (17.6)		5 (2.6)	
No one knows	381 (53.3)	98 (25.7)		31 (21.8)		8 (2.1)	
Someone knows and is phobic	143 (20.0)	43 (30.1)		31 (16.2)		9 (6.3)	
Coming-out status with current family			0.027∗		0.104		0.054
Living alone	390 (54.6)	107 (27.4)		65 (16.7)		8 (2.1)	
Someone knows and is supportive	141 (19.7)	22 (15.6)		23 (16.3)		5 (3.6)	
No one knows	127 (17.8)	28 (22.1)		24 (18.9)		4 (3.2)	
Someone knows and is phobic	57 (8.0)	17 (29.8)		17 (29.8)		5 (8.8)	
Internalized transphobia			0.001∗		<0.001∗		0.212
Low	80 (50.3)	14 (17.5)		7 (8.8)		4 (5.0)	
High	79 (49.7)	33 (41.8)		28 (35.9)		8 (10.3)	
Internalized homophobia			0.002∗		0.044∗		0.014∗
Low	310 (47.0)	56 (18.1)		43 (13.9)		4 (1.3)	
High	35 (53.0)	100 (28.6)		69 (19.8)		16 (4.6)	

^†^The first column presents column-wise percentages within each subcategory, whereas the prevalence values across rows represent row-wise percentages.

^‡^In the general population from the 9th Republic of Korea National Health and Nutrition Examination Survey (KNHANES, 2022–2024), 4.9% reported depressive symptoms, 4.6% suicidal ideation and 0.5% suicide attempts.

∗ Chi-square test was used and statistical significance was defined as *p* < 0.05, which is indicated with an asterisk (∗).

Table 2 presents findings related to the work environment. Managers and professionals reported a lower prevalence of depressive symptoms (19%, n = 27) compared to blue-collar workers (40.1%, n = 18). By workplace type, employees of international corporations and public institutions reported relatively low rates of depression (17.1% and 17.0%, respectively). The most common monthly income range was 2–3 million KRW, reported by 302 respondents (42.7%). Mental health outcomes were poorest among those in the lowest income group. Participants working 30–39 hours per week exhibited the lowest prevalence of depressive symptoms and suicide attempts (14.1%, n = 20). Larger workplaces and longer job tenure were associated with lower rates of depressive symptoms. Participants who experienced unwanted outing reported higher rates of suicide attempts.

**Table 2:** Work-related characteristics of responders and the prevalence of depressive symptoms, suicidal ideation and suicide attempt in each group

	Total	Depressive symptom		Suicidal ideation		Suicide attempt	
	*n* (% of total)	*n* (row %)	*p*	*n* (row %)	*p*	*n* (row %)	*p*
Total	720 (100%)	177 (24.6)		129 (17.9)		22 (3.1)	
Occupation			0.026∗		0.084		0.322
Manager and professionals	142 (19.7)	27 (19.0)		27 (19.0)		4 (2.8)	
White collar	390 (54.2)	93 (23.9)		58 (14.9)		9 (2.3)	
Pink collar	144 (20.0)	39 (27.1)		33 (23.1)		6 (4.2)	
Blue collar	44 (6.1)	18 (40.1)		11 (25.0)		3 (6.8)	
Place of employment			0.390		0.003∗		0.922
Korean-owned private company	505 (70.1)	131 (25.9)		83 (16.4)		16 (3.2)	
International corporation	35 (4.9)	6 (17.1)		6 (17.1)		1 (2.9)	
School and university	52 (7.2)	11 (21.2)		13 (25.0)		1 (1.9)	
Government agency	27 (3.8)	5 (18.5)		12 (44.4)		0 (0)	
Public institution and state-owned enterprise	53 (7.4)	9 (17.0)		5 (9.4)		2 (3.8)	
NGO, religious organization or cooperative	48 (6.7)	15 (31.3)		10 (21.3)		2 (3.8)	
Type of contract			<0.001∗		<0.001∗		0.267
Regular employed	599 (83.2)	128 (21.4)		93 (15.5)		16 (2.7)	
Temporary worker	94 (13.1)	37 (39.4)		31 (33.0)		4 (4.3)	
Day-labor	27 (3.8)	12 (44.4)		5 (19.2)		2 (7.7)	
Monthly income (1,000 Korean won) †			0.001∗		<0.001∗		0.011∗
Under 1,000	38 (5.4)	19 (50.0)		16 (42.1)		4 (10.5)	
1,000∼2,000	55 (7.8)	21 (38.2)		16 (29.1)		3 (5.5)	
2,000∼3,000	302 (42.7)	58 (19.2)		55 (18.3)		12 (4.0)	
3,000∼4,000	182 (25.7)	43 (23.6)		22 (12.1)		1 (0.6)	
4,000∼5,000	70 (9.9)	21 (30.0)		10 (14.3)		0 (0)	
5,000∼	60 (8.5)	12 (20.0)		10 (16.7)		2 (3.3)	
Weekly working hour (hour)			0.001∗		0.003∗		0.136
<15	44 (6.1)	14 (31.8)		12 (27.3)		2 (4.6)	
15-29	54 (7.5)	17 (32.5)		13 (24.1)		3 (5.6)	
30-39	142 (19.7)	20 (14.1)		20 (14.1)		5 (3.5)	
40-52	426 (59.2)	104 (24.4)		66 (15.5)		8 (1.9)	
More than 52	54 (7.5)	22 (40.7)		18 (33.3)		4 (7.4)	
Shift work			0.890		0.080		0.019∗
No	616 (85.6)	152 (24.7)		104 (16.9)		15 (2.4)	
Yes	104 (14.4)	25 (24.0)		25 (24.0)		7 (6.7)	
Don't know	18 (2.5)	6 (33.3)		4 (22.2)		2 (11.1)	
Tenure (year)			0.005∗		<0.001∗		0.663
0-1	174 (24.2)	58 (33.3)		50 (28.7)		6 (3.5)	
1-5	362 (50.3)	84 (23.2)		50 (13.9)		9 (2.5)	
More than 5	184 (25.6)	35 (19.0)		29 (15.8)		7 (3.8)	
Job security			0.086		0.112		0.940
Secure	618 (85.8)	145 (23.5)		105 (17.0)		19 (3.1)	
Insecure	102 (14.2)	32 (31.4)		24 (23.5)		3 (2.9)	
Labor union			0.071		0.017∗		0.535
Union member	121 (16.8)	19 (15.7)		20 (16.5)		3 (2.5)	
Union established but not a member	94 (13.1)	22 (23.4)		12 (12.8)		2 (2.1)	
No labor union	412 (57.2)	113 (27.4)		70 (17.0)		12 (2.9)	
Don't know	92 (12.8)	23 (25.0)		27 (29.4)		5 (5.4)	
Coming-out in work			0.450		0.511		0.005∗
Coming out to more than 1 person	176 (24.5)	37 (21.0)		36 (20.5)		8 (4.6)	
Outed, unwanted	55 (7.7)	15 (26.3)		11 (20.0)		5 (9.1)	
Never coming-out	488 (67.9)	124 (25.4)		82 (16.8)		9 (1.8)	
Having experience of declining for a job, considering one's SGM identity			0.014∗		<0.001∗		<0.001∗
Yes, I have	163 (22.6)	52 (31.9)		45 (27.6)		15 (9.20)	
Never	557 (77.4)	125 (22.4)		84 (15.1)		7 (1.26)	

NGO, nongovernmental organization; SGM, sexual and gender minority.

^‡^The first column presents column-wise percentages within each subcategory, whereas the prevalence values across rows represent row-wise percentages.

^§^In the general population from the 9th Republic of Korea National Health and Nutrition Examination Survey (KNHANES, 2022–2024), 4.9% reported depressive symptoms, 4.6% suicidal ideation and 0.5% suicide attempts.

∗ Chi-square test was used and statistical significance was defined as *p* < 0.05, which is indicated with an asterisk (∗).

† 1,360 Korean won is equivalent to 1 US dollar.

### 3.2 Disparities in mental health, working conditions, and workplace violence

Table 3 summarizes aPR for mental health outcomes. Compared to the general population, SGM participants had a 4.31 times higher prevalence of depressive symptoms (95% CI: 3.70–4.99), a 3.60 times higher for suicidal ideation (95% CI: 3.00–4.27), and a 4.52 times higher for suicide attempts (95% CI: 2.83–6.85). The mental health outcome prevalence of the general population, used as the reference in Table 3, is presented in Supplementary Table S2.

**Table 3:** Age-standardized prevalence ratios (aPRs) and 95% confidence intervals (CI) of mental health status among sexual and gender minority workers compared with the general population

Mental health status	aPR (95% CI)
Depressive symptom	4.31 (3.70-4.99) ∗
Suicidal ideation	3.60 (3.00-4.27) ∗
Suicide attempts	4.52 (2.83-6.85) ∗

aPR, age-standardized prevalence ratio; CI, confidence interval.

^†^Mental health outcomes were referenced to the general population from the 9th Republic of Korea National Health and Nutrition Examination Survey (KNHANES, 2022–2024).

∗ Statistically significant, confidence intervals do not contain 1.

Table 4 compares the prevalence of adverse working conditions between SGM workers and the general workforce. Insecure employment was 1.52 times more prevalent among SGM workers (95% CI: 1.24–1.85), off-hours work concerns were 3.89 times (95% CI: 3.41–4.43), and lack of family time was 2.33 times more common (95% CI: 2.01–2.68). Workplace violence was 5.16-fold higher in the past month (95% CI: 4.51–5.89) and 27.62-fold higher in the past year (95% CI: 23.22–32.62); unwanted sexual attention and bullying were 22.93- (95% CI: 18.85–27.64) and 71.57-fold higher (95% CI: 57.09–88.62), respectively. The aPRs were generally higher among managers, professionals, and white-collar workers, except for past-year physical violence. For Table 4, the working conditions and workplace violence of the general working population used as the reference are shown in Supplementary Table S3.

**Table 4:** Age-standardized prevalence ratio (aPR) and 95% confidence intervals (CI) of hazard working conditions and workplace violence experienced by sexual and gender minority workers versus the general working population

	Total	aPR (95% CI)	
		Manager, professionals, and white collar	Blue and pink collar
Security of job			
Insecure job	1.52 (1.24-1.85) ∗	1.86 (1.41-2.40) ∗	1.63 (1.19-2.17) ∗
Work-life balance			
Poor work-life balance	1.17 (1.00-1.37) ∗	1.29 (1.06-1.55) ∗	1.10 (0.81-1.46)
Keep worrying about work during off-duty	3.89 (3.41-4.43) ∗	3.86 (3.32-4.46) ∗	3.73 (2.79-4.88) ∗
Difficult to spend enough time with family due to work	2.33 (2.01-2.68) ∗	2.17 (1.82-2.57) ∗	2.66 (1.99-3.46) ∗
Workplace violence, over last 1 month			
Verbal abuse	3.85 (3.20-4.58) ∗	4.79 (3.85-5.90) ∗	3.15 (2.22-4.35) ∗
Unwanted sexual attention	22.93 (18.85-27.64) ∗	40.37 (32.01-50.24) ∗	14.49 (9.77-20.68) ∗
Threat	11.40 (8.54-14.91) ∗	25.38 (18.29-34.31) ∗	5.30 (2.64-9.49) ∗
Insult	6.26 (5.18-7.51) ∗	8.10 (6.46-10.03) ∗	4.91 (3.36-6.93) ∗
Any of above	5.16 (4.51-5.89) ∗	6.02 (5.10-7.05) ∗	4.67 (3.66-5.89) ∗
Workplace violence, over last 1 year			
Physical violence	14.83 (9.17-22.66) ∗	16.38 (8.71-28.01) ∗	17.85 (7.69-35.18) ∗
Sexual harassment	28.21 (22.26-35.25) ∗	31.06 (23.52-40.24) ∗	23.91 (14.60-36.93) ∗
Bullying	71.57 (57.09-88.62) ∗	96.25 (72.89-124.71)∗	59.77 (39.38-86.97) ∗
Any of above	27.62 (23.22-32.62) ∗	31.08 (25.03-38.16) ∗	27.54 (20.30-36.52) ∗

^†^Working condition and workplace violence experience variables were referenced to the general workers from the 7th Korean Working Conditions Survey (2023).

∗ Statistically significant, confidence intervals do not contain 1.

### 3.3 Risk and protective factors for mental health

Table 5 presents logistic regression results for factors associated with depressive symptoms among SGM participants. Higher odds were observed for limited social support at work (OR = 2.57, 95% CI: 1.80–3.68), a negative workplace climate (OR = 3.87, 95% CI: 2.68–5.57), poor work-life balance (OR = 3.84, 95% CI: 2.61–5.64), workplace violence in the past month (OR = 3.62, 95% CI: 2.53–5.20) or past year (OR = 2.92, 95% CI: 1.96–4.36), and refraining from job applications due to SGM identity (OR = 1.62, 95% CI: 1.09–2.39). Although not statistically significant, participants who had not undergone gender-affirming surgery had higher odds of depression (OR = 2.62, 95% CI: 0.94–7.30).

**Table 5:** Odds ratios (OR) and 95% confidence intervals (CI) of depressive symptoms according to variables among the Korean sexual and gender minority workers

Work related and minority identity related variables		OR (95% CI)
Work-related stress		
Social support in work	Good	1
	Bad	2.57 (1.80-3.68) ∗
Workplace climate	Good	1
	Bad	3.87 (2.68-5.57) ∗
Work-life balance	Good	1
	Bad	3.84 (2.61-5.64) ∗
Keep worrying about work during off-duty	Without interference	1
	With interference	5.33 (3.67-7.76) ∗
Difficult to spend enough time with family due to work	Without interference	1
	With interference	4.09 (2.81-5.95) ∗
Workplace violence		
Over last 1 month, any of	None	1
	Having experience	3.62 (2.53-5.20) ∗
Over last 1 year, any of	None	1
	Having experience	2.92 (1.96-4.36) ∗
Security of job	Secure	1
	Insecure	1.35 (0.84-2.17)
Labor union	Union member	1
	Union established but not a member	1.78 (0.89-3.55)
	No labor union in company	2.02 (1.16-3.51) ∗
	Don't know	1.80 (0.89-3.63)
Weekly working hour	<15	2.94 (1.29-6.71) ∗
	15-29	2.92 (1.36-6.28) ∗
	30-39	1
	40-52	1.94 (1.15-3.29) ∗
	More than 52	4.18 (2.00-8.71) ∗
Experience of giving up on applying for a job due to SGM identity	None	1
	Having experience	1.62 (1.09-2.39) ∗
Coming-out in work	Coming out to more than 1 person	1
	Outed, unwanted	1.31 (0.65-2.65)
	Never coming-out	1.23 (0.81-1.88)
Family support and coming-out status		
Family of origin	Someone knows and is supportive	1
	No one knows	1.58 (1.02-2.46) ∗
	Someone knows and is phobic	2.01 (1.19-3.39) ∗
Current family	Someone knows and is supportive	1
	Living alone	2.04 (1.22-3.39) ∗
	No one knows	1.47 (0.78-2.76)
	Someone knows and is phobic	2.37 (1.13-4.98) ∗
Internalized phobia		
Internalized homophobia	Low	1
	High	1.77 (1.21-2.58) ∗
Internalized transphobia	Low	1
	High	3.18 (1.49-6.80) ∗
Gender-affirming care		
Gender-affirming hormone therapy	Having now	1
	Having experience, now quitting	1.07 (0.28-4.03)
	Never	0.99 (0.41-2.40)
Gender-affirming surgery	Having (any surgery)	1
	Never	2.62 (0.94-7.30)
Gender identity	Cisgender men	1
	Cisgender women	1.38 (0.91-2.10)
	Transmasculine	1.46 (0.66-3.26)
	Transfeminine	0.75 (0.33-1.71)
	Nonbinary	2.42 (1.39-4.22) ∗

SGM, sexual and gender minority.

^†^Covariates in the model include age and occupation.

∗ Statistically significant, confidence intervals do not contain 1.

## 4 Discussion

SGM workers are subject to minority stress—a chronic form of stress stemming from their marginalized social status 10. Building upon this framework, L.M. Diamond introduced the concept of social safety, which refers to a sense of belonging and perceived protection within a social environment [33]. In the absence of such safety, individuals remain hypervigilant to potential threats. Stigmatized individuals may anticipate hostility, while feeling socially safe allows for emotional relaxation, which positively influences mental health. Therefore, improving the mental health of SGM individuals requires not only reducing overt discrimination and violence but also cultivating environments that foster safety and inclusion.

In this study, SGM workers in the Republic of Korea reported significantly higher levels of workplace violence compared to the general workforce, and many refrained from applying for jobs due to their identity. Furthermore, only 24.5% of participants had voluntarily disclosed their identity to at least one colleague. These findings suggest that many SGM workers in the Republic of Korea do not perceive their workplaces as socially safe environments.

As shown in Table 1, Table 2, poorer mental health outcomes were associated with adverse conditions such as precarious employment, lower income, and extremely short or long working hours. These findings align with existing literature on the relationship between occupational conditions and depressive symptoms [[34], [35], [36], [37]]. Notably, SGM employees at international corporations reported lower levels of depressive symptoms, possibly reflecting the presence of inclusive workplace policies that are often lacking in domestic Korean firms.

The aPRs for depressive symptoms, suicidal ideation, and suicide attempts (Table 3) ranged from 3.60 to 4.52. Although elevated, these values were somewhat lower than those reported in a 2017 study, which found aPRs ranging from 5.08 to 37.65 in a broader sample of sexual minorities not restricted to the employed population [21]. This discrepancy may reflect the “healthy worker effect,” wherein individuals who are employed tend to have better overall health than the general population [38]. However, these figures still suggest a substantially elevated risk. Suicide, in particular, is the most serious outcome and shows a high prevalence in SGM populations. Although the range is wide due to the limited sample sizes of some studies, a recent review reported that the prevalence of suicidality among the SGM workers reached up to 64.8% [39]. Prior research has also found that social discrimination, including work-related discrimination and harassment, is associated with increased prevalence of suicidal ideation and suicide attempts [40,41].

Employment instability was also more prevalent among SGM workers, consistent with prior findings showing that SGM individuals are more likely to hold precarious jobs [15,42]. Work-related interference was more common among SGM participants than in the general workforce. Many Korean SGM workers may fear negative consequences from workplace disclosure and may attempt to compensate by maintaining high work performance. This interpretation aligns with findings from prior qualitative studies conducted in the Republic of Korea [43]. Poor work-life balance and work-related interference were both associated with significantly increased odds of depressive symptoms.

The elevated aPRs for workplace violence found in this study are consistent with prior research documenting high levels of harassment among SGM populations. For example, 35% of cisgender SGM employees and 57% of transgender and nonbinary workers have reported identity-based workplace harassment [13,14]. Gender minorities may be particularly vulnerable, as their identities are often more visible. Even when SGM individuals choose not to disclose their identity, they may still be targeted due to nonconformity with socially normative gender expressions.

In our analysis, workplace violence and job stress were both significantly associated with depressive symptoms. These findings underscore the importance of reducing workplace violence as a key strategy to improve SGM workers' mental health. Importantly, such efforts also benefit cisgender heterosexual workers, contributing to a healthier and safer work environment overall.

Although a positive organizational climate does not guarantee SGM inclusivity, social support and favorable workplace conditions were protective against depressive symptoms. Union membership was not significantly related to depressive symptoms, suggesting that labor unions in the Republic of Korea may not currently function as safe or supportive spaces for SGM workers. Unlike international findings [44,45], workplace coming-out status was not significantly associated with mental health in this study, likely reflecting low inclusivity in Korean workplaces where disclosure is made to few trusted colleagues.

Gender minorities frequently face barriers not only in entering but also in maintaining employment, which increases their vulnerability to mental health problems. In our logistic regression analysis, having refrained from applying for a job due to SGM identity was significantly associated with depressive symptoms. Giving up job opportunities due to identity was particularly common among gender minorities (22.6% of SGM workers; 61.0% of transgender participants, n = 50) (Supplementary Table S1). Although the association with depressive symptoms was not statistically significant in this study, previous research suggests that gender-affirming care may support mental health [46]. However, in the Republic of Korea, lack of insurance coverage and paid leave creates financial and institutional barriers, making continued employment during transition challenging.

Workplace violence is a key risk factor for poor mental health, highlighting the need for nondiscriminatory work environments. Supportive relationships—from family, colleagues, and supervisors—enhance psychological safety, and clear, consistent signals of safety at interpersonal, organizational, and national levels are essential to mitigate chronic stress and address persistent mental health challenges among SGM workers.

This study examined the excessive mental health burden among Korean SGM workers and the workplace-related risks to their mental health. Several limitations should be acknowledged. First, self-report questionnaires may have introduced recall bias, though structured instruments were used where possible.

Second, the online survey was limited to those with link access, introducing selection bias. Given that national surveys in the Republic of Korea do not adequately address SGM issues, and that social stigma may discourage honest responses, recruiting a representative sample remains a challenge. In addition, working condition questions may have been challenging for precarious or informal workers, contributing to higher dropout rates.

Third, transgender and nonbinary individuals often face significant barriers from the outset of the job search process. As a result, those included in the study may represent a limited subset of this population, and the labor and mental health challenges of this group may be underrepresented in our findings. Further research is needed to better understand the labor market experiences and mental health, particularly those marginalized by gender identity.

Despite these limitations, this study provides several important contributions. To our knowledge, it is the first large-scale investigation in the Republic of Korea to link labor conditions and mental health among SGM workers. Second, it highlights the stark disparities in mental health between SGM and cisgender heterosexual workers, reinforcing the need for inclusive labor and health policies. Third, it identifies risk and protective factors associated with the mental health of SGM workers through subgroup-specific analyses, informing targeted interventions.

## 5 Conclusion

Excessive mental health challenges among Korean SGM workers are linked to multiple factors, including hazardous working conditions and workplace violence. Addressing these issues requires a comprehensive approach that promotes SGM inclusivity across interpersonal relationships, organizational practices, and national policies.

## CRediT authorship contribution statement

**Munyoung Yang:** Writing – original draft, Software, Methodology, Investigation, Formal analysis, Data curation, Conceptualization. **Hanul Kim:** Writing – review & editing, Validation, Resources, Formal analysis, Data curation. **Keonhee Cho:** Writing – review & editing, Formal analysis, Data curation, Conceptualization. **Sejung Kim:** Writing – review & editing, Supervision, Resources, Formal analysis, Data curation. **Sinyoung Yang:** Validation, Methodology, Investigation, Data curation. **Soyeong Park:** Writing – review & editing, Data curation, Conceptualization. **Suejin Yeo:** Writing – review & editing, Data curation, Conceptualization. **Hyoung-Ryoul Kim:** Writing – review & editing, Project administration, Methodology, Investigation, Conceptualization.

## Author disclosure statement

This work was supported by the Republic of Korea Institute of Labor Safety and Health (KILSH) and the Legal Assistance Network for Queer Workers. Both organizations are nongovernmental organizations (NGOs) engaged in social movements to promote workers' health rights and civil human rights. While some of the researchers involved in this study are members of these NGOs, all participated in the research independently. Neither organization was directly involved in conducting the research. These NGOs are funded by donations from their members and do not receive financial support from governments or corporations.

## Statement on the use of AI

During the preparation of this manuscript, the authors did not use any generative AI or AI-assisted technologies for idea generation, data analysis, or manuscript drafting. Only minor language and grammar editing assistance was obtained from an AI-based tool. The authors have carefully reviewed and revised all content and take full responsibility for the originality and accuracy of the manuscript.

## Funding

This work was supported by small-scale research grants from the Korea Institute of Labor Safety and Health (KILSH) and the Legal Assistance Network for Queer Workers. The NGOs had no role in the study design, data analysis, or any other aspect of the research, and there was no intervention or influence from either organization in the research process.

## Conflicts of interest

All authors have no conflicts of interest to declare.
