# Supplementary material for: Mental Health Challenges and Workplace Risk Factors Among Sexual and Gender Minority Workers in Korea
Source: Saf Health Work. 2026 May 5;17(3):331–9. doi: 10.1016/j.shaw.2026.04.005 (PMC13650336; doi:10.1016/j.shaw.2026.04.005)
Supplement: Supplementary file 1 [file mmc1.docx]

Supplementary table S1. Experience of giving up on applying for a job due to sexual and gender minority identity

|  |  | None  N (%) | Having experience  N (%) |
| --- | --- | --- | --- |
| Total |  | 557 (77.4) | 163 (22.6) |
| Gender identity | Transgender | 32 (39.0) | 50 (61.0) |
|  | Nonbinary | 56 (71.8) | 22 (28.2) |
|  | Cisgender | 469 (83.8) | 91 (16.2) |
| Gender expression | Consistent with legal gender | 456 (82.5) | 97 (17.5) |
|  | Inconsistent with legal gender^a^ | 101 (60.5) | 66 (39.5) |

*Degree of incongruence varies across individuals

Supplementary table S2. The mental health outcome prevalence of general population, the reference of Table 3.

| Age group | Mental health outcome prevalence (%) | | |
| --- | --- | --- | --- |
|  | Depressive symptom | Suicidal ideation | Suicide attempts |
| 19-29 | 6.47 | 7.33 | 0.67 |
| 30-39 | 5.62 | 4.11 | 0.77 |
| 40-49 | 4.56 | 3.54 | 0.25 |
| 50-59 | 3.93 | 3.64 | 0.49 |
| 60-64 | 3.41 | 4.54 | 0.33 |
| † Mental health outcomes were from the 9th Korea National Health and Nutrition Examination Survey (KNHANES, 2022–2024) | | | |

Supplementary table S3. The hazard working conditions and workplace violence experience of general working population, the reference of Table 4.

|  |  |  | Prevalence of hazard working conditions and workplace violence experience (%) | | |
| --- | --- | --- | --- | --- | --- |
|  |  | Age | Total | Manager, professionals,  and White collar | Blue and  Pink collar |
| Security of job | |  |  |  |  |
|  | Insecure job | 19-29 | 15.2 | 8.5 | 23.4 |
|  |  | 30-39 | 6.3 | 4.8 | 9.7 |
|  |  | 40-49 | 7.7 | 4.2 | 13.7 |
|  |  | 50-59 | 12.9 | 7.2 | 16.8 |
|  |  | 60-64 | 21.5 | 13.0 | 23.3 |
| Work-life balance | |  |  |  |  |
|  | Poor work-life balance | 19-29 | 19.6 | 16.3 | 23.6 |
|  |  | 30-39 | 17.8 | 15.6 | 22.9 |
|  |  | 40-49 | 19.7 | 15.8 | 26.2 |
|  |  | 50-59 | 19.4 | 13.8 | 23.0 |
|  |  | 60-64 | 21.6 | 13.4 | 23.4 |
|  | Keep worrying about work  during off-duty | 19-29 | 6.8 | 7.3 | 6.3 |
|  |  | 30-39 | 8.8 | 9.3 | 7.6 |
|  |  | 40-49 | 9.6 | 9.7 | 9.4 |
|  |  | 50-59 | 10.6 | 11.1 | 10.3 |
|  |  | 60-64 | 9.8 | 10.2 | 9.8 |
|  | Difficult to spend  enough time  with family due to work | 19-29 | 8.4 | 10.0 | 6.8 |
|  |  | 30-39 | 12.6 | 12.5 | 12.7 |
|  |  | 40-49 | 12.3 | 12.0 | 12.9 |
|  |  | 50-59 | 13.1 | 13.4 | 12.9 |
|  |  | 60-64 | 10.7 | 7.8 | 11.4 |
| Workplace violence, over last 1 month | |  |  |  |  |
|  | Verbal abuse | 19-29 | 5.6 | 3.9 | 7.5 |
|  |  | 30-39 | 4.3 | 3.4 | 6.5 |
|  |  | 40-49 | 4.3 | 3.5 | 5.7 |
|  |  | 50-59 | 5.1 | 3.5 | 6 |
|  |  | 60-64 | 6.0 | 2.7 | 6.7 |
|  | Unwanted sexual attention | 19-29 | 0.8 | 0.2 | 1.4 |
|  |  | 30-39 | 0.7 | 0.5 | 1.1 |
|  |  | 40-49 | 0.6 | 0.4 | 0.8 |
|  |  | 50-59 | 0.9 | 0.5 | 1.2 |
|  |  | 60-64 | 0.4 | 0.2 | 0.5 |
|  | Threat | 19-29 | 0.8 | 0.2 | 1.4 |
|  |  | 30-39 | 0.5 | 0.4 | 0.9 |
|  |  | 40-49 | 0.9 | 0.7 | 1.3 |
|  |  | 50-59 | 1.0 | 0.6 | 1.2 |
|  |  | 60-64 | 1.1 | 0.8 | 1.2 |
|  | Insult | 19-29 | 3.0 | 1.9 | 4.3 |
|  |  | 30-39 | 2.3 | 1.9 | 3.0 |
|  |  | 40-49 | 3.3 | 2.7 | 4.2 |
|  |  | 50-59 | 2.9 | 2.2 | 3.4 |
|  |  | 60-64 | 4.3 | 1.9 | 4.8 |
|  | Any of above | 19-29 | 7.0 | 5.1 | 9.1 |
|  |  | 30-39 | 5.5 | 4.6 | 7.8 |
|  |  | 40-49 | 5.9 | 4.9 | 7.5 |
|  |  | 50-59 | 6.6 | 4.5 | 8.0 |
|  |  | 60-64 | 7.8 | 3.7 | 8.7 |
| Workplace violence, over last 1 year | |  |  |  |  |
|  | Physical violence | 19-29 | 0.2 | 0.1 | 0.3 |
|  |  | 30-39 | 0.2 | 0.2 | 0.1 |
|  |  | 40-49 | 0.2 | 0.1 | 0.3 |
|  |  | 50-59 | 0.5 | 0.2 | 0.6 |
|  |  | 60-64 | 0.3 | 0 | 0.4 |
|  | Sexual harassment | 19-29 | 0.6 | 0.6 | 0.7 |
|  |  | 30-39 | 0.3 | 0.3 | 0.3 |
|  |  | 40-49 | 0.3 | 0.2 | 0.5 |
|  |  | 50-59 | 0.7 | 0.4 | 0.5 |
|  |  | 60-64 | 0.4 | 0.1 | 0.4 |
|  | Bullying | 19-29 | 0.1 | 0.1 | 0.2 |
|  |  | 30-39 | 0.2 | 0.1 | 0.4 |
|  |  | 40-49 | 0.2 | 0.2 | 0.2 |
|  |  | 50-59 | 0.3 | 0.3 | 0.2 |
|  |  | 60-64 | 0.2 | 0.2 | 0.1 |
|  | Any of above | 19-29 | 0.9 | 0.7 | 1.2 |
|  |  | 30-39 | 0.6 | 0.5 | 0.8 |
|  |  | 40-49 | 0.6 | 0.5 | 0.8 |
|  |  | 50-59 | 1.2 | 0.8 | 1.5 |
|  |  | 60-64 | 0.7 | 0.3 | 0.8 |
| †Working condition and workplace violence experience were from the 7th Korean Working Conditions Survey(2023) | | | | | |
